# Fast Alignment of SINS for Marching Vehicles Based on Multi-Vectors of Velocity Aided by GPS and Odometer

**DOI:** 10.3390/s18010137

**Published:** 2018-01-05

**Authors:** Chunxi Zhang, Longjun Ran, Lailiang Song

**Affiliations:** School of Instrumentation Science and Opto-electronics Engineering, Beihang University, Beijing 100191, China; zhangchunxi@buaa.edu.cn (C.Z.); ranlongjun@buaa.edu.cn (L.R.)

**Keywords:** vehicle-mounted SINS, fast alignment for marching base, multi-vector attitude determination, error propagation characteristic, GPS and Odometer

## Abstract

In the strap-down inertial navigation system (SINS), the initial attitude matrix is acquired through alignment. Though there were multiple valid methods, alignment time and accuracy are still core issues, especially regarding the condition of the motion carrier. Inspired by the idea of constructing nonlinear vectors by velocity in a different coordinate frame, this paper proposes an innovative alignment method for a vehicle-mounted SINS in motion. In this method, the core issue of acquiring the attitude matrix is to calculate the matrix between the inertial frame and the initial body frame, which can be constructed through the nonlinear velocity vectors’ information from the GPS and the odometer at different moments, which denominate the multi-vector attitude determination. The possibility of collinearity can easily be avoided by a turning movement. The characteristic of propagation of error is analyzed in detail, based on which an improved method is put forward to depress the effect of random noise. Compared with the existing alignment methods, this method does not use the measurement information of accelerometers. In order to demonstrate its performance, the method is compared with the two-position alignment method and the traditional two-stage alignment method. Simulation and vehicle-based experiment results show that the proposed alignment method can establish an attitude reference in 100 s with an azimuth error of less than 0.06°, and that the accuracy does not have a strong correlation with the accelerometer.

## 1. Introduction

Alongside the significant advances in the inertial sensors and navigation technologies, strap-down inertial navigation systems (SINS) have reduced in structure complexity and become a key member of the navigation system [1]. The strap-down inertial navigation system, simplified as SINS, is fixedly installed in the carrier, and the gyroscopes and accelerometers immediately sense and detect the dynamic characteristics of the carrier. The navigation solution is achieved through a mathematical platform, which is different from the gimbaled inertial navigation system. The SINS is an independent navigation system that has been used in a wide range of applications, covering the navigation of marines, vehicles, missiles, and aircraft [2]. 

It is widely known that the state update of the SINS is achieved based on numerical integration [3]; therefore, it is necessary to know the initial navigation parameters, including velocity, position, and attitude for navigation calculation. The initial alignment of the SINS is mainly about the determination of the initial attitude matrix between the body frame and the navigation frame, since the velocity and position can be easily obtained by external reference [4]. Initial alignment is one of the core technologies for the SINS; the requirements of initial alignment are high accuracy and short time [1]. It is urgently expected to achieve an accurate alignment within a short time; however, it is difficult and contradictory to achieve such an endpoint, especially for the SINS on a moving base [5,6,7].

The existing alignment methods typically comprise two steps: coarse alignment and fine alignment. The goal of a coarse alignment is to provide a roughly known initial attitude within a short time, while the fine alignment mainly drives the misalignment angles to zero [8]. An analytic method using the known gravity and Earth rate signals in the local level frame and the measurements obtained by using accelerometers and gyros to obtain the system attitude directly is often used for coarse alignment when the SINS is in stationary conditions [9]. Meanwhile, optimal estimation methods based on filter are used to realize the fine alignment [10]. 

To improve the self-alignment performance of the SINS in stationary bases, the multi-position alignment method was put forward, as it can improve the observability of the SINS by changing the position or equivalently by rotating vehicle body [2,11]. In contrast, the two-position method is mostly defined in the static base. It can evaluate some bias and factor errors of the inertial sensors, and achieve higher alignment accuracy. However, two parameters must be considered in order to employ the method effectively: the minimum number of positions required to obtain the best alignment, and the optimum configuration for a given multi-position alignment [11]. 

Besides alignment in stationary conditions, a substantial amount of research work about initial alignment on a moving base has been launched. Transfer alignment is one of the most important methods to align inertial navigation systems on moving bases [12,13,14,15]. The alignment of a slave system with respect to a master reference is called transfer alignment, which is widely used for the SINS in ships and planes for its speed and high accuracy compared with other alignment methods [16]. Choosing a reasonable matching method is one of the key points of transfer alignment. The transfer alignment method has entered a rapid development and widely used period after the “velocity plus attitude” matching method, which was first put forward by Kain and Clouiter in 1989 [16]. Transfer alignment models include linear and nonlinear models, which respectively correspond with the small and large misalignment angles [17]. 

In addition to transfer alignment, some other research studies about alignment methods on an in-motion base are also done. Gravity and Earth rational rate cannot be accurately measured due to the high frequency noise in the shacking base; as a result, some methods to eliminate the noise from the outputs of inertial sensors are proposed to achieve higher accuracy [18,19,20,21]. Since the outputs of inertial sensors installed on the board directly are vulnerable to external interference, many researchers devoted their energy to improving the performance of the initial alignment with external disturbance. 

Since 2000, the articles from IXBlue Company claimed that their newest product—Octans—can fulfill alignment within five minutes under any swinging condition by observing the gravitational apparent motion. As a result, various alignment methods have been proposed based on the idea of tracking gravitational apparent motion [17,21,22,23,24,25,26]. Similar to the analytic method, the basic idea of both methods for obtaining initial attitude is to construct noncollinear vectors by tracking gravitational apparent motion in an inertial frame [26,27,28]. There are two problems caused by the alignment mechanism: how to avoid collinear possibility between two vectors, and how to recognize the gravitational apparent motion. 

Based on the idea of constructing noncollinear vectors, many research studies have attempted to achieve the alignment in marching bases with much more simple methods. With the aid of velocity from the GPS, Dr. Peter M. G. Silson and Dr. Simon Jordan introduced a method to accomplish the alignment with the velocity vectors obtained from the GPS and the integrated accelerations [27]. For the low-cost internal navigation system (INS), Han, Songlai introduced an alignment method with two-stage Kalman filtering; with the coarse alignment and the fine alignment, the proposed alignment approach can complete the initial alignment more quickly and more accurately [29]. For the application in landed carriers, Chang, Lubin introduced an attitude estimation-based alignment method, which could attenuate the disturbance in the odometer to a certain extent, and with a low-pass finite impulse response digital filter, the novel method does have a better performance compared with the attitude determination-based method [30]. However, there is still much space to shorten the alignment time and improve the accuracy. The information from the GPS and the odometer could be combined organically to achieve much better performance in the initial alignment of the SINS.

In this paper, a novel alignment method for the marching vehicle is proposed based on the idea of matrix decomposition. In this method, the constant matrix from the initial body frame to the inertial frame is calculated through vectors constructed by the velocity from the GPS in the navigation frame, and the velocity from the odometer in the body frame. Using the outputs of gyros tracks the changes of body frame, and using the position information (latitude and longitude) from GPS and the alignment time calculates the matrix from the inertial frame to the navigation frame. Compared with the alignment method based on the tracking gravitational apparent motion, this method can easily avoid the collinear possibility by a turning movement. Compared with the current alignment for a marching vehicle [31], this method can fulfill alignment in a short time, and does not need the coarse alignment stage before marching. Compared with the existing alignment methods, this method does not use the measurement information of accelerometers. The outstanding characteristic of this method is that the accuracy of the azimuth is superior, especially with low-precision inertial sensors, and the initial alignment is completed totally in motion. Through the simulation, vehicle experiments, and the comparison with the two-position alignment method, the new alignment mechanism can achieve higher azimuth accuracy in much shorter time.

## 2. New Alignment Mechanism Based on the GPS and the Odometer

### 2.1. Coordinate Frame Definitions

The coordinate frames used in this paper are defined as follows:

- The e frame is the Earth-fixed coordinate frame, whose origin is the center of the Earth. The $x_{e}$ axis is in the equatorial plane and points to the prime meridian, and the $z_{e}$ axis is parallel to the rotation axis of the Earth. The $y_{e}$ axis completes the right-handed coordinate system.
- The $e_{0}$ frame is also the Earth-fixed coordinate frame, whose origin is the center of the Earth. The $x_{e0}$ axis is in the equatorial plane, and points to the meridian of the vehicle’s position at the beginning of initial alignment, and the $z_{e0}$ axis is parallel to the rotation axis of the Earth. The $y_{e0}$ axis completes the right-handed coordinate system.
- The $i_{0}$ frame is formed by fixing the $e_{0}$ frame at the beginning of alignment in inertial space.
- The n frame is the instantaneous navigation coordinate frame, which is the local level coordinate frame. The $x_{n}$ axis points to the east, the $y_{n}$ axis points to the north, and the $z_{n}$ axis points upwards.
- The b frame is the instantaneous body coordinate frame, which is defined as the common “Right–Forward–Up” frame.
- The $i_{b0}$ frame is formed by fixing the b frame at the beginning of alignment in the inertial space.

### 2.2. Constructing Nonlinear Vectors by Turning Movement

The alignment method of the SINS in this paper, which is aided by the GPS and the odometer, is designed for a marching vehicle on land. The crucial point, whose first step is to construct nonlinear vectors, is how to figure out the matrix between the $i_{0}$ frame and the $i_{b0}$ frame.

**Figure 1:**
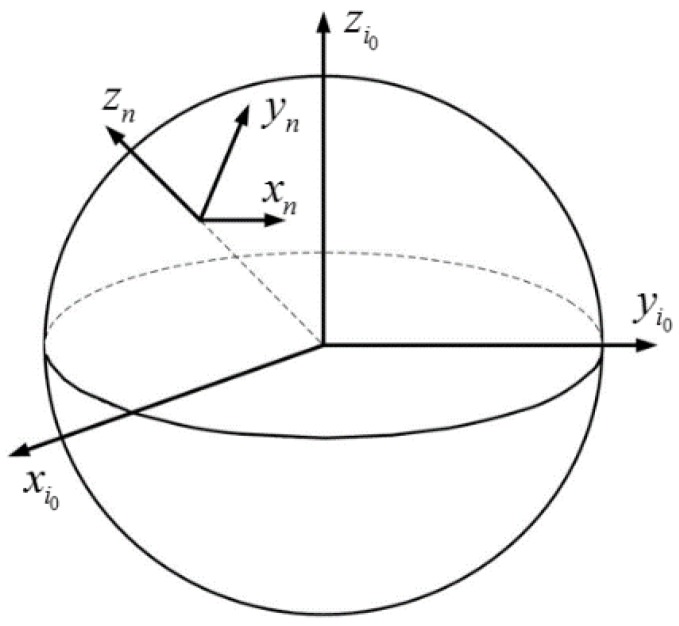
The $i_{0}$ frame and the n frame on the Earth.

The $i_{0}$ frame and the n frame on the Earth are shown as Figure 1. When the vehicle moves along a straight line for a short period of time, the n frame can be recognized as unchangeable but for its origin, thus the velocity of vehicle in the n frame is parallel in this period of time, whose projections in the $i_{0}$ frame are collinear consequently. Then, how can the nonlinear vectors be obtained in a short period of time?

**Figure 2:**
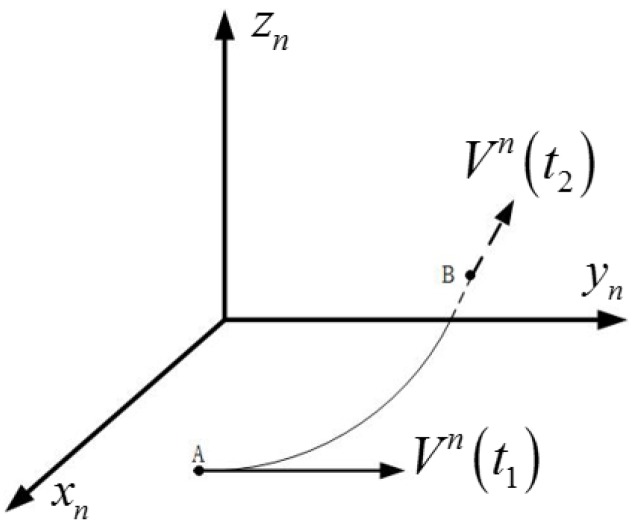
Turning movement in the n frame.

Obviously, if the vehicle moves with a turning at a certain moment, the vehicle’s velocity in the n frame before turning is not parallel to that after turning. It is assumed that: the vehicle at position A moves with a ground speed of $V^{n}\left(t_{1}\right)$ at $t_{1}$ moment, and the direction of the velocity is along the $y_{n}$ axis, as shown in Figure 2; then, the vehicle moves along the smooth curve from position A to position B at $t_{2}$ moment, where its ground speed is recorded as $V^{n}\left(t_{2}\right)$, and the motion directs to the reverse of the axis $x_{n}$. It is obvious that $V^{n}\left(t_{1}\right)$ and $V^{n}\left(t_{2}\right)$ are not parallel, and the projections of them in the $i_{0}$ frame are nonlinear vectors as a consequence.

It is worth noting that the nonlinear vectors are obtained by a turning movement with the angle of 90°, as shown in Figure 2. It is quite understandable that $V^{n}\left(t_{1}\right)$ and $V^{n}\left(t_{2}\right)$ will be parallel again if the turning angle becomes 180°, which should be avoided. Theoretically, if only the turning angle is larger than 0° and smaller than 180°, the nonlinear vectors can be obtained. In fact, in order to cut down the calculation error and depress the interference from measurement errors of velocity, the turning angle should have a large discrepancy relative to 0° and 180°.

### 2.3. Alignment Algorithm

The attitude matrix $C_{b}^{n}\left(t\right)$ changes with time when the vehicle is in motion. In order to obtain it, the matrix $C_{b}^{n}\left(t\right)$ can be decomposed into two parts, as follows [32]:

(1) $$C_{b}^{n}\left(t\right)=C_{i_{0}}^{n}\left(t\right)C_{b}^{i_{0}}\left(t\right)$$

where $C_{N}^{M}$ is the attitude matrix between N and M.

The matrix $C_{i_{0}}^{n}\left(t\right)$ in Equation (1) can be calculated as follows [33]:

(2) $$C_{i_{0}}^{n}\left(t\right)=C_{e}^{n}\left(t\right){\left(C_{e}^{n_{0}}\right)}^{T}C_{e_{0}}^{n_{0}}C_{i_{0}}^{e_{0}}\left(t\right)$$

The matrix $C_{e}^{n}\left(t\right)$ $C_{e}^{n_{0}}$ $C_{e_{0}}^{n_{0}}$ can be acquired through the latitude and longitude of the vehicle, while the matrix $C_{i_{0}}^{e_{0}}\left(t\right)$ can be determined constantly through the alignment time. Therefore, the specific expression of matrix $C_{i_{0}}^{n}$ is as follows:

(3) $$C_{i_{0}}^{n}\left(t\right)=\left[\begin{array}{ccc} -\sin\left[\Delta \lambda +\omega_{ie}t\right] & \cos\left[\Delta \lambda +\omega_{ie}t\right] & 0\\ -\sin L_{t}\cos\left[\Delta \lambda +\omega_{ie}t\right] & -\sin L_{t}\sin\left[\Delta \lambda +\omega_{ie}t\right] & \cos L_{t}\\ \cos L_{t}\cos\left[\Delta \lambda +\omega_{ie}t\right] & \cos L_{t}\sin\left[\Delta \lambda +\omega_{ie}t\right] & \sin L_{t} \end{array}\right]$$

where Δλ can be determined by $\Delta \lambda =\lambda_{t}-\lambda_{0}$, and $\lambda_{0}$ is the meridian of the vehicle’s position at the beginning of initial alignment; $L_{t}$ and $\lambda_{t}$ are the latitude and longitude at the current time t, respectively; $\lambda_{0}$, $L_{t}$ and $\lambda_{t}$ can be obtained from the GPS, or the $L_{t}$ and $\lambda_{t}$ can be computed through $\lambda_{0}$ and the velocity from the GPS; and $\omega_{ie}$ refers to the Earth rotation angular velocity.

The matrix $C_{b}^{i_{0}}\left(t\right)$ indicates the transformation relation between the body frame and the initial inertial coordinate frame. Furthermore, $C_{b}^{i_{0}}\left(t\right)$ can be decomposed as follows [32]:

(4) $$C_{b}^{i_{0}}\left(t\right)=C_{i_{b0}}^{i_{0}}\left(t\right)C_{b}^{i_{b0}}\left(t\right)$$

With the information from the gyroscope, the matrix $C_{b}^{i_{b0}}\left(t\right)$ in Equation (4) can be determined constantly through the attitude-updating algorithm [24]:

(5) $${\dot{C}}_{b}^{i_{b0}}\left(t\right)=C_{b}^{i_{b0}}\left(t\right)\left[\omega_{i_{b0}b}^{b}\left(t\right)\times \right]$$

where the $\omega_{i_{b0}b}^{b}$ is the gyro measurement value, and [•×] represents the skew-symmetric matrix of vector •.

According to Equations (1) and (5), the problem of solving $C_{b}^{n}\left(t\right)$ in the SINS alignment can be attributed to the computing of $C_{i_{b0}}^{i_{0}}$. Inspired by the idea of tracking gravitational apparent motion to construct noncollinear vectors [22,25], $C_{i_{b0}}^{i_{0}}$ can be obtained if the projection of the identical physical vector is known in the $i_{0}$ frame and the $i_{b0}$ frame synchronously. Then, the specific expression of $C_{i_{b0}}^{i_{0}}$ will be deduced in detail.

In the SINS aided by the GPS and the odometer, the velocity of a vehicle in the n frame can be obtained from the GPS, while the odometer can provide the velocity of a vehicle in the b frame through misalignment compensation. The misalignment angles between the odometer and the b-frame, computed as $\alpha ={\left[\begin{array}{ccc} \alpha_{\theta } & \alpha_{\gamma } & \alpha_{\psi } \end{array}\right]}^{T}$, are calibrated in advance. The velocity of a vehicle in the n frame and the b frame are defined respectively as follows:

(6) $$\{\begin{array}{c} V^{n}\left(t\right)={\left[\begin{array}{ccc} V_{E}^{n} & V_{N}^{n} & V_{U}^{n} \end{array}\right]}^{T}\\ V^{b}\left(t\right)={\left[\begin{array}{ccc} V_{x}^{b} & V_{y}^{b} & V_{z}^{b} \end{array}\right]}^{T}=C_{b}^{m}•{\left[\begin{array}{ccc} 0 & V^{m} & 0 \end{array}\right]}^{T} \end{array}$$

where $V_{E}^{n}$, $V_{N}^{n}$, and $V_{U}^{n}$ denote the vehicle’s velocity in the n frame pointing east, north, and upwards, respectively; $V_{x}^{b}$, $V_{y}^{b}$, and $V_{z}^{b}$ denote the vehicle’s velocity in the b frame, and $V^{m}$ is the velocity from the odometer pointing forwards; and $C_{b}^{m}$ denotes the transform matrix between the odometer and the b-frame. In most cases, the $C_{b}^{m}$ could be simplified as follows, considering that $\alpha_{\theta }$, $\alpha_{\gamma }$ and $\alpha_{\psi }$ are always small quantities.

$$C_{b}^{m}=\left[\begin{array}{ccc} 1 & -\alpha_{\psi } & \alpha_{\gamma }\\ \alpha_{\psi } & 1 & -\alpha_{\theta }\\ -\alpha_{\gamma } & \alpha_{\theta } & 1 \end{array}\right]$$

It is obvious that the velocity’s projection of a vehicle in the $i_{0}$ frame ($V^{i_{0}}\left(t\right)$) can be obtained by $V^{n}\left(t\right)$, while that in the $i_{b0}$ frame, ($V^{i_{b0}}\left(t\right)$) can be calculated through $V^{b}\left(t\right)$ as follows:

(7) $$\{\begin{array}{c} V^{i_{0}}\left(t\right)=C_{n}^{i_{0}}\left(t\right)V^{n}\left(t\right)\\ V^{i_{b0}}\left(t\right)=C_{b}^{i_{b0}}\left(t\right)V^{b}\left(t\right) \end{array}$$

where $C_{n}^{i_{0}}\left(t\right)={\left(C_{i_{0}}^{n}\left(t\right)\right)}^{T}$ can be obtained by Equation (3); and $C_{b}^{i_{b0}}\left(t\right)$ can be obtained by Equation (5).

The relationship between $V^{i_{0}}\left(t\right)$ and $V^{i_{b0}}\left(t\right)$ is explicit, as follows:

(8) $$V^{i_{b0}}\left(t\right)=C_{i_{0}}^{i_{b0}}V^{i_{0}}\left(t\right)$$

where $C_{i_{0}}^{i_{b0}}={\left(C_{i_{b0}}^{i_{0}}\right)}^{T}$. The matrix can be computed through a series of noncollinear vectors, which can be acquired by choosing a different time $t_{j}$ in the alignment stage. Therefore, the formula is as follows:

(9) $$V_{j}^{i_{b0}}\left(t\right)=C_{i_{0}}^{i_{b0}}V_{j}^{i_{0}}\left(t\right)\;j=1,2,\cdots m$$

In order to compute the optimal solution of $C_{i_{0}}^{i_{b0}}$, construct the indicator function as follows [24]:

(10) $$J^{*}(C_{i_{0}}^{i_{b0}})=\frac{1}{2}\sum_{j=1}^{m}w_{j}{\left|V_{j}^{i_{b0}}-C_{i_{0}}^{i_{b0}}V_{j}^{i_{0}}\right|}^{2}=\min$$

where $w_{j}$ is the weighting coefficient, and the sum of squares $\left|V_{j}^{i_{b0}}-C_{i_{0}}^{i_{b0}}V_{j}^{i_{0}}\right|$ can be calculated as follows:

(11) $$\begin{array}{ll} {\left|V_{j}^{i_{b0}}-C_{i_{0}}^{i_{b0}}V_{j}^{i_{0}}\right|}^{2} & \;={\left(V_{j}^{i_{b0}}-C_{i_{0}}^{i_{b0}}V_{j}^{i_{0}}\right)}^{T}(V_{j}^{i_{b0}}-C_{i_{0}}^{i_{b0}}V_{j}^{i_{0}})\\ & \;=\left[{\left(V_{j}^{i_{b0}}\right)}^{T}-{\left(V_{j}^{i_{0}}\right)}^{T}{\left(C_{i_{0}}^{i_{b0}}\right)}^{T}\right](V_{j}^{i_{b0}}-C_{i_{0}}^{i_{b0}}V_{j}^{i_{0}})\\ & \;={\left|V_{j}^{i_{b0}}\right|}^{2}-{\left(V_{j}^{i_{b0}}\right)}^{T}C_{i_{0}}^{i_{b0}}V_{j}^{i_{0}}-{\left(V_{j}^{i_{0}}\right)}^{T}{\left(C_{i_{0}}^{i_{b0}}\right)}^{T}{\tilde{V}}_{i}^{r}+{\left(V_{j}^{i_{0}}\right)}^{T}{\left(C_{i_{0}}^{i_{b0}}\right)}^{T}C_{i_{0}}^{i_{b0}}V_{j}^{i_{0}}\\ & \;={\left|V_{j}^{i_{b0}}\right|}^{2}+{\left|V_{j}^{i_{0}}\right|}^{2}-2{\left(V_{j}^{i_{b0}}\right)}^{T}C_{i_{0}}^{i_{b0}}V_{j}^{i_{0}} \end{array}$$

Therefore, Equation (10) is equivalent to the formula below:

(12) $$\begin{array}{ll} J^{*}(C_{i_{0}}^{i_{b0}}) & =\frac{1}{2}\sum_{j=1}^{m}w_{j}{\left|V_{j}^{i_{b0}}-C_{i_{0}}^{i_{b0}}V_{j}^{i_{0}}\right|}^{2}\\ & =\frac{1}{2}\sum_{j=1}^{m}w_{j}({\left|V_{j}^{i_{b0}}\right|}^{2}+{\left|V_{j}^{i_{0}}\right|}^{2})-\sum_{j=1}^{m}w_{j}{\left(V_{j}^{i_{b0}}\right)}^{T}C_{i_{0}}^{i_{b0}}V_{j}^{i_{0}}=\min \end{array}$$

The indicator function can be reconstructed as follows:

(13) $$J(C_{i_{0}}^{i_{b0}})=\sum_{j=1}^{m}w_{j}{\left(V_{j}^{i_{b0}}\right)}^{T}C_{i_{0}}^{i_{b0}}V_{j}^{i_{0}}=\max$$

The simplified computing of the indicator function is stated as Equation (14):

(14) $$\begin{array}{c} J(C_{i_{0}}^{i_{b0}})\;=\mathrm{tr}(\left[\begin{array}{c} w_{1}{\left(V_{1}^{i_{b0}}\right)}^{T}\\ w_{2}{\left(V_{2}^{i_{b0}}\right)}^{T}\\ \vdots \\ w_{m}{\left(V_{m}^{i_{b0}}\right)}^{T} \end{array}\right]C_{i_{0}}^{i_{b0}}\left[\begin{array}{cccc} V_{1}^{i_{0}} & V_{2}^{i_{0}} & \cdots & V_{m}^{i_{0}} \end{array}\right])=\mathrm{tr}(C_{i_{0}}^{i_{b0}}\left[\begin{array}{cccc} V_{1}^{i_{0}} & V_{2}^{i_{0}} & \cdots & V_{m}^{i_{0}} \end{array}\right]\left[\begin{array}{c} w_{1}{\left(V_{1}^{i_{b0}}\right)}^{T}\\ w_{2}{\left(V_{2}^{i_{b0}}\right)}^{T}\\ \vdots \\ w_{m}{\left(V_{m}^{i_{b0}}\right)}^{T} \end{array}\right])\\ J(C_{i_{0}}^{i_{b0}})\;=\mathrm{tr}(C_{i_{0}}^{i_{b0}}\sum_{j=1}^{m}w_{j}V_{j}^{i_{0}}{\left(V_{j}^{i_{b0}}\right)}^{T})=\max \end{array}$$

With the reconstructed indicator function as Equation (14), the matrix $\left[\sum_{j=1}^{m}w_{j}V_{j}^{i_{0}}{\left(V_{j}^{i_{b0}}\right)}^{T}\right]$ can be decomposed to $UDV^{T}$, which is known as singular value decomposition, supposing $\det\left(\sum_{j=1}^{m}w_{j}V_{j}^{i_{0}}{\left(V_{j}^{i_{b0}}\right)}^{T}\right)>0$. The optimal attitude matrix can be computed as follows:

(15) $$C_{i_{0}}^{i_{b0}}=UV^{T}$$

Generally speaking, $C_{i_{b0}}^{i_{0}}$ in Equation (15) cannot be orthogonal for the calculated errors caused by measurement errors of the GPS and the odometer, and this $C_{i_{b0}}^{i_{0}}$ should be further orthogonalized as follows [22]:

(16) $${\left(C_{i_{b0}}^{i_{0}}\right)}_{o}=C_{i_{b0}}^{i_{0}}{\left[{\left(C_{i_{b0}}^{i_{0}}\right)}^{T}C_{i_{b0}}^{i_{0}}\right]}^{-\frac{1}{2}}$$

where (•)*_o_* represents the result of matrix • through orthogonalization.

Ultimately, substituting Equations (3), (4), and (16) into Equation (1), the initial alignment can be fulfilled.

It deserves much attention that $V^{i_{0}}\left(t_{j}\right)$ and $V^{i_{0}}\left(t_{j}\right)$ should be noncollinear, which can be guaranteed by the turning movement of the vehicle, as analyzed in Section 3.1. It is obvious that accelerometer measurements are not used in this alignment method, which is the different from the existing alignment method.

## 3. Error Analysis and the Improved Alignment Method

### 3.1. Error Analysis

The novel alignment method is detailed as presented above, and it is essential to figure out the influence factor of the initial errors. The real-time propagation error of the alignment procedure based on a multi-vector may be somewhat complicated, and it may be more important to get the elements relating to the misalignment. To obtain the error equations, similar to the coarse inertial alignment method, two different moments are selected arbitrarily to execute the alignment errors.

Without loss of generality, two specified moments, $T_{l}$ and $T_{m}$, are selected (assuming $T_{l}<T_{m}$), and the simplified calculation of $C_{i_{0}}^{i_{b0}}$ can be determined as follows, consulting the coarse alignment method based on the inertial frame [28].

(17) $$C_{i_{0}}^{i_{b0}}={\left(C_{i_{b0}}^{i_{0}}\right)}^{T}={\left[\begin{array}{c} {\left[V^{i_{b0}}\left(T_{l}\right)\right]}^{T}\\ {\left[V^{i_{b0}}\left(T_{l}\right)\times V^{i_{b0}}\left(T_{m}\right)\right]}^{T}\\ {\left[V^{i_{b0}}\left(T_{l}\right)\times V^{i_{b0}}\left(T_{m}\right)\times V^{i_{b0}}\left(T_{l}\right)\right]}^{T} \end{array}\right]}^{-1}\left[\begin{array}{c} {\left[V^{i_{0}}\left(T_{l}\right)\right]}^{T}\\ {\left[V^{i_{0}}\left(T_{l}\right)\times V^{i_{0}}\left(T_{m}\right)\right]}^{T}\\ {\left[V^{i_{0}}\left(T_{l}\right)\times V^{i_{0}}\left(T_{m}\right)\times V^{i_{0}}\left(T_{l}\right)\right]}^{T} \end{array}\right]$$

According to the character of the orthorhombic matrix, the new expression of $C_{i_{b0}}^{i_{0}}$ is as follows:

(18) $$C_{i_{b0}}^{i_{0}}={\left(C_{i_{0}}^{i_{b0}}\right)}^{T}=C^{i_{0}}•C_{i_{b0}}$$

where the $C^{i_{0}}$ and $C_{i_{b0}}$ are defined in Equation (19):

(19) $$\left\{ \begin{array}{l} C^{i_{0}}=\left[\begin{array}{ccc} V^{i_{0}}\left(T_{l}\right), & V^{i_{0}}\left(T_{l}\right)\times V^{i_{0}}\left(T_{m}\right), & V^{i_{0}}\left(T_{l}\right)\times V^{i_{0}}\left(T_{m}\right)\times V^{i_{0}}\left(T_{l}\right) \end{array}\right]\\ C_{i_{b0}}={\left[\begin{array}{ccc} V^{i_{b0}}\left(T_{l}\right), & V^{i_{b0}}\left(T_{l}\right)\times V^{i_{b0}}\left(T_{m}\right), & V^{i_{b0}}\left(T_{l}\right)\times V^{i_{b0}}\left(T_{m}\right)\times V^{i_{b0}}\left(T_{l}\right) \end{array}\right]}^{-1} \end{array}\right.$$

Therefore, the attitude matrix at $T_{m}$ can be rewritten as Equation (20):

(20) $$C_{b}^{n}\left(T_{m}\right)=C_{i_{0}}^{n}\left(T_{m}\right)C_{b}^{i_{0}}\left(T_{m}\right)=C_{i_{0}}^{n}\left(T_{m}\right)•C_{i_{b0}}^{i_{0}}•C_{b}^{i_{b0}}\left(T_{m}\right)=C_{i_{0}}^{n}\left(T_{m}\right)•C^{i_{0}}•C_{i_{b0}}•C_{b}^{i_{b0}}\left(T_{m}\right)$$

Taking account of the short interval between $T_{l}$ and $T_{m}$, as the initial alignment is typically fulfilled in minutes or shorter, it is rational to assume that the longitude and latitude remain constant throughout the alignment procedure. Thus, there exists an approximate equality of $C_{i_{0}}^{n}\left(T_{m}\right)\approx C_{i_{0}}^{n}\left(T_{l}\right)$. Therefore, the attitude matrix at $T_{m}$ is finally determined as Equation (21):

(21) $$C_{b}^{n}\left(T_{m}\right)=C_{i_{0}}^{n}\left(T_{m}\right)•C^{i_{0}}•C_{i_{b0}}•C_{b}^{i_{b0}}\left(T_{m}\right)=C^{n}•C_{i_{b0}}•C_{b}^{i_{b0}}\left(T_{m}\right)$$

where $C^{n}=\left[\begin{array}{ccc} V^{n}\left(T_{l}\right), & V^{n}\left(T_{l}\right)\times V^{n}\left(T_{m}\right), & V^{n}\left(T_{l}\right)\times V^{n}\left(T_{m}\right)\times V^{n}\left(T_{l}\right) \end{array}\right]$.

Assuming that the variation of the Euler angles of the body frame at $T_{l}$ and $T_{m}$ are Δθ, Δγ and Δψ, the following equation is acceptable.

(22) $$\Delta C=C_{b(T_{m})}^{b(T_{l})}=C_{i_{b0}}^{b}\left(T_{l}\right)C_{b}^{i_{b0}}\left(T_{m}\right)=\left[\begin{array}{ccc} \cos(\Delta \gamma )\cos(\Delta \psi )+\sin(\Delta \gamma )\sin(\Delta \psi )\sin(\Delta \theta ) & -\cos(\Delta \gamma )\sin(\Delta \psi )+\sin(\Delta \gamma )\cos(\Delta \psi )\sin(\Delta \theta ) & -\sin(\Delta \gamma )\cos(\Delta \theta )\\ \sin(\Delta \psi )\cos(\Delta \theta ) & \cos(\Delta \psi )\cos(\Delta \theta ) & \sin(\Delta \theta )\\ \sin(\Delta \gamma )\cos(\Delta \psi )-\cos(\Delta \gamma )\sin(\Delta \psi )\sin(\Delta \theta ) & -\sin(\Delta \gamma )\sin(\Delta \psi )-\cos(\Delta \gamma )\cos(\Delta \psi )\sin(\Delta \theta ) & \cos(\Delta \gamma )\cos(\Delta \theta ) \end{array}\right]$$

In order to simplify the calculation process, the angles Δθ and Δγ can be assumed to be zero, taking account of the motion forms in most cases. Thus, the transformation matrix ΔC can be rewritten as follows:

(23) $$\Delta C=\left[\begin{array}{ccc} \cos(\Delta \psi ) & -\sin(\Delta \psi ) & 0\\ \sin(\Delta \psi ) & \cos(\Delta \psi ) & 0\\ 0 & 0 & 1 \end{array}\right]$$

Based on Equations (21) and (23), it is understandable to yield Equation (24):

(24) $$C_{b}=C_{i_{b0}}C_{b}^{i_{b0}}\left(T_{m}\right)={\left[\begin{array}{ccc} {\left(\Delta C\right)}^{T}V^{b}\left(T_{l}\right), & \left({\left(\Delta C\right)}^{T}V^{b}\left(T_{l}\right)\right)\times V^{b}\left(T_{m}\right), & \left({\left(\Delta C\right)}^{T}V^{b}\left(T_{l}\right)\right)\times V^{b}\left(T_{m}\right)\times \left({\left(\Delta C\right)}^{T}V^{b}\left(T_{l}\right)\right) \end{array}\right]}^{-1}$$

Therefore, the explicit expression of $C_{b}^{n}\left(t_{2}\right)$ can be calculated through the following equation:

(25) $$C_{b}^{n}\left(T_{m}\right)=C_{i_{0}}^{n}\left(T_{m}\right)•C^{i_{0}}•C_{i_{b0}}•C_{b}^{i_{b0}}\left(T_{m}\right)=C^{n}C_{b}$$

The attitude angle between the n frame and the b frame is defined as $\phi ={\left[\begin{array}{ccc} \theta & \gamma & \varphi \end{array}\right]}^{T}$, where θ, γ, and φ denote pitch, roll, and yaw respectively. It is widely known that ϕ can be expressed as follows:

(26) $$\{\begin{array}{c} \sin\theta =C_{32}\\ \tan\gamma =\frac{-C_{31}}{C_{33}}\\ \tan\varphi =\frac{-C_{12}}{C_{22}} \end{array}$$

where $C_{MN}$ is the element of $C_{b}^{n}\left(T_{m}\right)$ at row M and column N.

According to Equations (21), (23), (24), and (26), the attitude angle can be computed as follows:

(27) $$\{\begin{array}{c} \sin\theta =\frac{V_{U}^{n}\left(T_{m}\right)}{V_{y}^{b}\left(T_{m}\right)}\\ \tan\gamma =\frac{V_{y}^{b}\left(T_{m}\right)V_{U}^{n}\left(T_{l}\right)-V_{y}^{b}\left(T_{l}\right)V_{U}^{n}\left(T_{m}\right)\cos\left(\Delta \psi \right)}{V_{E}^{n}\left(T_{l}\right)V_{N}^{n}\left(T_{m}\right)-V_{E}^{n}\left(T_{m}\right)V_{N}^{n}\left(T_{l}\right)}\\ \tan\varphi =-\frac{V_{E}^{n}\left(T_{m}\right)}{V_{N}^{n}\left(T_{m}\right)} \end{array}$$

Taking the time derivative on both sides of Equation (27), it gives:

(28) $$\{\begin{array}{c} d\theta =\frac{V_{y}^{b}\left(T_{l}\right)dV_{U}^{n}\left(T_{m}\right)-V_{U}^{n}\left(T_{m}\right)dV_{y}^{b}\left(T_{m}\right)}{{\left(V_{y}^{b}\left(T_{m}\right)\right)}^{2}\cos\theta }\\ d\gamma =\frac{PdQ+QdP}{\left[{\left(V_{E}^{n}\left(T_{m}\right)V_{N}^{n}\left(T_{l}\right)-V_{E}^{n}\left(T_{l}\right)V_{N}^{n}\left(T_{m}\right)\right)}^{2}\right]•{\left(\sec\gamma \right)}^{2}}\\ d\varphi =-\frac{V_{N}^{n}\left(T_{m}\right)dV_{E}^{n}\left(T_{m}\right)-V_{E}^{n}\left(T_{m}\right)dV_{N}^{n}\left(T_{m}\right)}{{\left(V_{N}^{n}\left(T_{m}\right)\right)}^{2}•{\left(\sec\varphi \right)}^{2}} \end{array}$$

where $P=\left(V_{E}^{n}\left(T_{m}\right)V_{N}^{n}\left(T_{l}\right)-V_{E}^{n}\left(T_{l}\right)V_{N}^{n}\left(T_{m}\right)\right)$, $Q=V_{U}^{n}\left(T_{m}\right)V_{y}^{b}\left(T_{l}\right)\cos\left(\Delta \psi \right)-V_{U}^{n}\left(T_{l}\right)V_{y}^{b}\left(T_{m}\right)$, and d(•) denotes differential coefficient of •.

The following equations are understandable, considering that the value of pitch and roll are small.

(29) $$\{\begin{array}{c} \sin\theta \approx 0,\cos\theta \approx 1\\ \sin\gamma \approx 0,\cos\gamma \approx 1\\ V_{U}^{n}\left(t\right)\approx 0 \end{array}$$

The relationship among $V^{n}\left(t\right)$, $V^{b}\left(t\right)$, and ϕ is obviously as follows:

(30) $$\{\begin{array}{c} V_{E}^{n}\left(t\right)=-V_{y}^{b}\left(t\right)\cos\theta \sin\varphi \\ V_{N}^{n}\left(t\right)=V_{y}^{b}\left(t\right)\cos\theta \cos\varphi \\ V_{U}^{n}\left(t\right)=V_{y}^{b}\left(t\right)\sin\theta \end{array}$$

Substituting Equations (29) and (30) into Equation (28) yields:

(31) $$\{\begin{array}{c} d\theta =\frac{dV_{U}^{n}\left(T_{m}\right)}{V_{y}^{b}\left(T_{m}\right)}\\ d\gamma =\frac{dV_{U}^{n}\left(T_{m}\right)}{V_{y}^{b}\left(T_{m}\right)•\tan\left(\Delta \psi \right)}-\frac{dV_{U}^{n}\left(T_{l}\right)}{V_{y}^{b}\left(T_{l}\right)•\sin\left(\Delta \psi \right)}\\ d\varphi =-\frac{\cos\varphi •dV_{E}^{n}\left(T_{m}\right)}{V_{y}^{b}\left(T_{m}\right)}-\frac{\sin\varphi •dV_{N}^{n}\left(T_{m}\right)}{V_{y}^{b}\left(T_{m}\right)} \end{array}$$

Alignment errors are mainly affected by measurement errors of velocity from the GPS, the turning angle, and the vehicle’s velocity participating in calculation. Alignment errors are also affected by the odometer’s measurement errors, which provide the forward velocity as $V_{y}^{b}\left(t\right)$. It is understandable that Δψ should be far from 0° and 180°, and high speed leads to small errors.

### 3.2. Improved Method

According to the analysis in Section 3.1, the main error resource is the measurement errors of speed from the GPS, mostly with the character of stochastic error. It is universally known that integration can depress the effect of random noise; based on this, an improved method to calculate $C_{i_{b0}}^{i_{0}}$ is put forward.

Taking the time integral on both sides of Equation (7) yields:

(32) $$\{\begin{array}{c} u=\int V^{i_{0}}\left(t\right)dt=\int C_{n}^{i_{0}}\left(t\right)V^{n}\left(t\right)dt\\ \upsilon =\int V^{i_{b0}}\left(t\right)dt=\int C_{b}^{i_{b0}}\left(t\right)V^{b}\left(t\right)dt \end{array}$$

The indicator function of Equation (14) can be rewritten as follows:

(33) $$J(C_{i_{0}}^{i_{b0}})=\mathrm{tr}(C_{i_{0}}^{i_{b0}}\sum_{j=1}^{m}w_{j}u_{j}{\left(\upsilon_{j}\right)}^{T})=\max$$

Through singular value decomposition, the optimal attitude matrix $C_{i_{0}}^{i_{b0}}$ can be computed in the same way as Equation (15). This $C_{i_{0}}^{i_{b0}}$ should also be further orthogonalized, as in Equation (16).

## 4. Simulation and Vehicle Test

### 4.1. Simulation

#### 4.1.1. Simulation Settings

In order to demonstrate the alignment method proposed in this paper, an experiment is carried out and the motive trajectory could be generated through simulating the actual motion of a vehicle. The vehicle is assumed to have translational movement with a turning at an arbitrary moment among the motion. Firstly, the vehicle is assumed to be heading forward at the speed of 50 m/s at beginning; in the next 25 s, there are kinematics e.g., acceleration and deceleration, pitching and rolling motion (approximately within one degree); then the vehicle starts turning at the speed of 10 m/s; a turning with 50° will be finished in 10 s, i.e., the yaw angle changes from 70° to 120° ($\Delta \psi =50^{{}^{\circ}}$); the vehicle begins accelerating with the unchangeable acceleration for 10 s; then, with several motions, such as pitching and rolling; all of the kinematics are terminated at the 50th s, when the speed of the vehicle is 60 m/s; then, the vehicle moves along a straight line at this velocity, which lasts for the rest of the time. The whole simulation time can be changed considering the requirements of different methods. 

The traditional two-position alignment method can evaluate sensor errors, and in most cases, it can represent a relatively high alignment accuracy of the SINS with specified inertial sensor precision. The process of the two-position method is different from the new method, while it can be the benchmark for the novel alignment method. Therefore, we conducted the simulation as a comparison reference for the new method. The process of two-position alignment is described as: firstly, keep still for 30 s to fulfill the coarse alignment, which can obtain the initial attitude with large errors. Then, turning 90° changes the position only by angular movement, among which a liner Kalman filter is used to drive the misalignment angles to zero.

The initial position of alignment is set to $\lambda_{0}$ = 116.34° and $L_{0}$ = 39.98°, and the initial attitude is assumed as $\phi_{0}={\left[\begin{array}{ccc} 1^{{}^{\circ}} & 1^{{}^{\circ}} & 70^{{}^{\circ}} \end{array}\right]}^{T}$. The sample period of gyro and accelerometer is 0.001 s or 1 millisecond, while the update rate of the velocity from the GPS and the odometer is 10 Hz. The sensor errors are defined in Table 1. The random errors of the GPS in different directions are recognized as irrelevant.

With the above setting, the theoretical outputs of the gyros, the accelerometer, the GPS, and the odometer can be generated by back-stepping the navigation solution of the SINS. When the errors in Table 1 are added to these theoretical outputs, the data sensors output can be produced. In order to evaluate the alignment accuracy, the theoretical values of attitude are used as references. The difference between alignment attitude and the references are regarded as alignment errors.

#### 4.1.2. Simulation Results and Analysis

##### Error Analysis Validation

The alignment accuracy is mainly related to the measurement errors of the velocity information obtained through the GPS and the odometer, as stated in Section 3.1. To testify to the analysis results, a comparison could be conducted between the “alignment error” and the “calculation error”, which represent the error of the alignment process and the error computed in Equation (31), respectively. The raw alignment method stated in Section 2.3, without the integration of the velocity information, is conducted with the situation in Section 4.1.1. 

**Figure 3:**
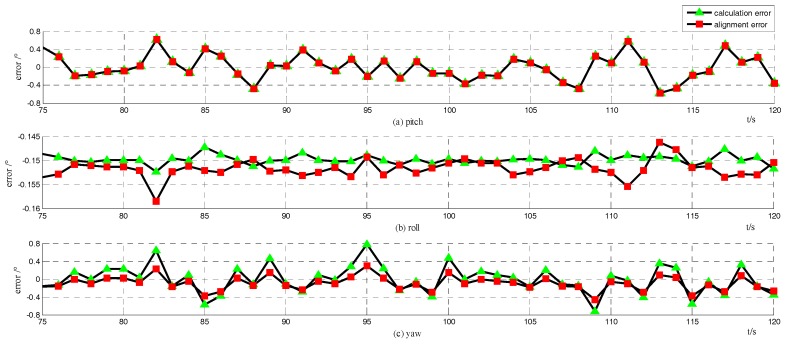
Attitude error for raw method: the green triangles are the calculation errors by Equation (31); the red squares denote the alignment errors.

The simulation results are shown in Figure 3, in which (a)–(c) denote the alignment errors of pitch, roll, and yaw, respectively, and the red squares denote the alignment errors from 75 to 120 s, while the green triangles are the calculation errors in Equation (31). The abscissa represents time, whose unit is second, while the vertical coordinates represents errors with respect to the unit of degree. The statistics of the alignment errors and calculation errors are shown in Table 2. There is little difference between the “alignment error” and the “calculation error”. The simulation results illustrate that the error analysis, deduced in Section 3.1, is reasonable. 

##### Alignment Mechanism Validation

**Figure 4:**
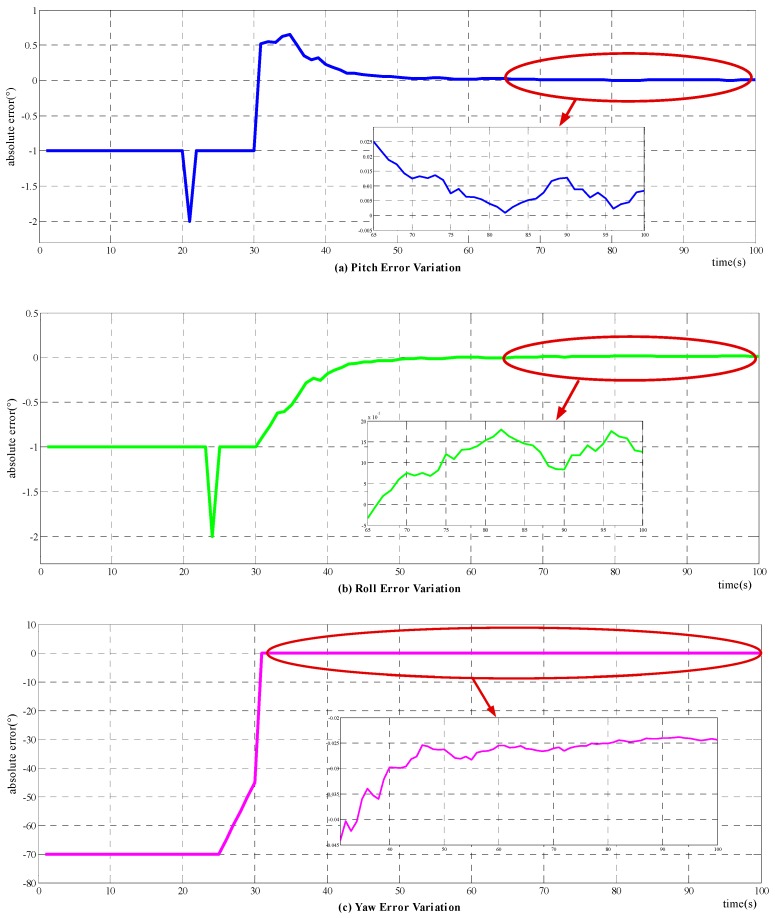
Variation of attitude error with novel alignment method: (**a**) the error variation of pitch; (**b**) the error variation of roll; and (**c**) the error variation of yaw.

Based on the simulation condition set above, a simulation experiment is carried out. The results of the novel method are shown in Figure 4, in which (a)–(c) denote the alignment errors of pitch, roll, and yaw, respectively, and the accompanying diagram shows the detailed convergence process of the corresponding attitude angle. The abscissa represents time in seconds, while the vertical coordinate represents attitude errors in degrees. The output attitude is not available until the 30th second of the alignment duration. Before the output attitude is available at the 30th s, the pitch, roll, and yaw errors just represent the actual attitude of the carrier. The spikes in (a) and (b) reflect the changing of pitch and roll in the marching of the carrier, as stated in Section 4.1.1. The simulation results illustrate that the novel method can fulfill the initial alignment for motion vehicles in 100 s, with horizontal attitude error about 0.015°, and azimuth error about 0.03°. Comparing with the simulation results of the raw method, as shown in Figure 4, the improved method does have a higher alignment accuracy.

**Figure 5:**
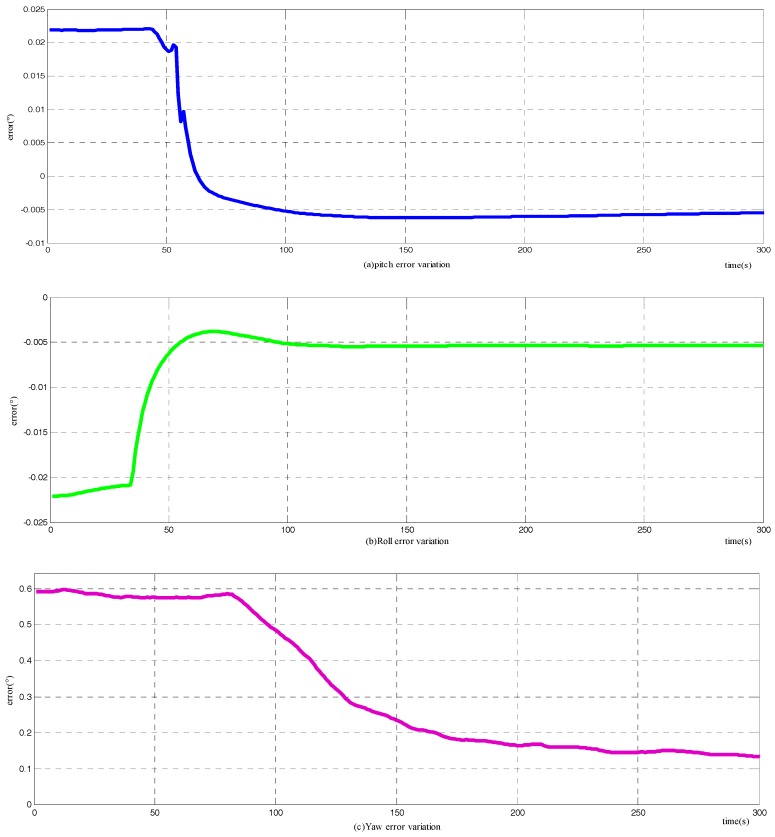
Variation of attitude error among two-position alignment stage: (**a**) the error variation of pitch; (**b**) the error variation of roll; and (**c**) the error variation of yaw.

The frequently-used two-position alignment method is carried out to compare with the novel method, and the variation of attitude errors are presented in Figure 5. The convergence time of the horizontal attitude angles is about 100 s, while it requires approximately 250 s for the azimuth to accomplish its constriction. However, the two-position alignment method does have a higher horizontal angle accuracy, and practically the same azimuth precision as the novel method.

**Figure 6:**
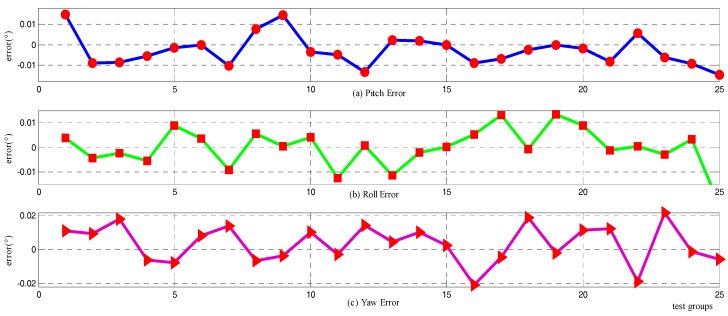
Repeatability of the novel alignment method: (**a**) the error discreteness of pitch; (**b**) the error discreteness of roll; (**c**) the error discreteness of yaw.

**Figure 7:**
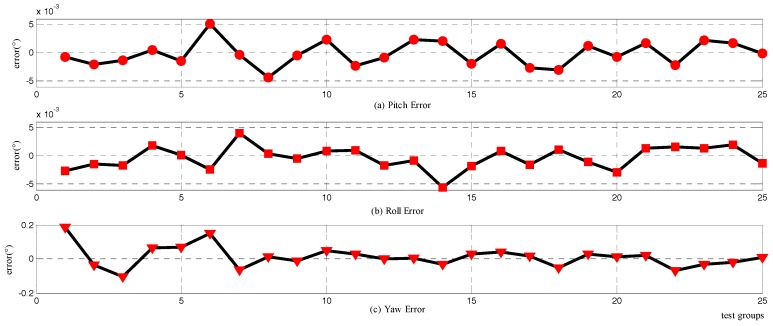
Repeatability of the two-position alignment method: (**a**) The error discreteness of pitch; (**b**) the error discreteness of roll; and (**c**) the error discreteness of yaw.

Without loss of generality, the simulation of the novel alignment method and the two-position method is promoted 25 times to illustrate the validity and the repeatability of the alignment results. The simulation conditions vary accordingly through changing the maneuver manners of the vehicles, e.g., the pitching or rolling amplitude, the turning angle, the forward speed, etc. The attitude errors are at the 300th s are regarded as alignment errors for the two-position alignment method, while the attitude errors are at the 100th s for the novel method. Therefore, the statistic characteristics of the simulation results are presented in Figure 6 and Figure 7 for the novel method and the two-position alignment method, respectively.

For the two-position alignment method, the attitude errors are definitely controlled within 0.01° for the horizontal angle and 0.4° for the azimuth (peak to peak). Taking account of the inertial sensors, including gyros and accelerometers with biases of 0.1°/h and 500 μg, respectively, the alignment results practically achieve the theoretical precision. Meanwhile, as the novel alignment method does not have strong correlation with the accuracy of gyros and accelerometers, the attitude errors converge to 0.03° for the horizontal angle and 0.04° for the azimuth, and the alignment results are mostly affected by the turning angle, the velocity of the GPS, and the odometer.

Comparing the alignment time and accuracy of these two methods, the novel procedure, as presented at Section 3, can fulfill the entire alignment process in 100 s, which is much shorter than the traditional two-position method. The horizontal results are slightly worse, while the azimuth does have more excellent performance than the two-position method, supposing the sensor errors exhibited in Table 1. In consideration of the simulation condition, the improved method can fulfill the initial alignment totally in motion, and do have superior azimuth accuracy and a much shorter time, which markedly improves the maneuverability of the vehicle.

### 4.2. Vehicle Test

The vehicle test was conducted to validate the actual performance of the novel alignment mechanism. The navigation system in the experiment is manufactured by our own research group, and the parameters of inertial sensors are similar with Table 1. The GNSS receiver is purchased from NovAtel, including the GPS-702-GG and the OEM615 as receiver. The velocity accuracy of the receiver can achieve 0.03 m/s (RMS), and the horizonal position accuracy is 1.5 m (RMS). The odometer is purchased from the state enterprise and it was utilized in many fields. The output of the odometer is pulse signal, and the scale factor parameter is under 0.2%. 

As attitude reference, the high-precision SINS was chosen to conduct the experiment, and the main characteristics are shown in Table 3. Considering the superior pose-maintaining performance in the navigation phase, the attitude output of the high-precision navigation system could be the reference of the novel method.

**Figure 8:**
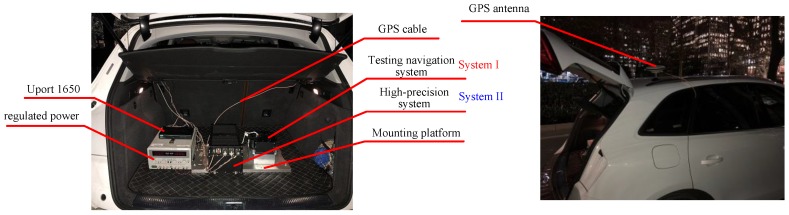
Devices mounted in the testing car for alignment experiment. UPort 1650 (MOXA, Taiwan): USB-to-serial converter.

The experiment program can be described as follows:

(a) The testing navigation system (simplified as System I) and the reference system (simplified as System II) were mounted in the testing car, as shown in Figure 8. The receiver of the GPS is integrated in the navigation system, the information from the GPS can be shared by System I and System II. The velocity information from the odometer is pulse signal, which can be collected by System I immediately.
(b) The misalignment of the two systems could be obtained through optical sighting, as these two systems had installed the optical prism. The misalignment must be compensated when processing the output data.
(c) System I and System II were launched simultaneously and with a strick on the mounting platform. System II would start its alignment and navigation procedure in normal mode. The testing carrier could moving out after the alignment of System II finished. All of the data from System I and System II, including the inertial sensor, the GPS, and the odometer, should be saved in the computer for further processing.

**Figure 9:**
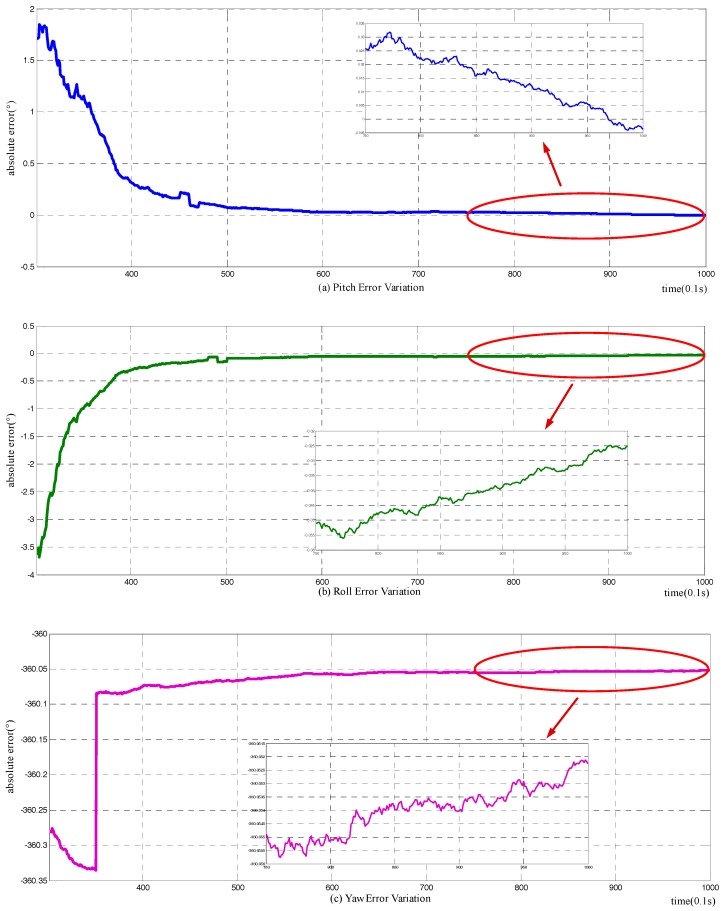
Attitude errors of the novel alignment method in a moving vehicle test: (**a**) the error curves of pitch; (**b**) the error curves of roll; and (**c**) the error curves of yaw.

With the assistance of synchronous processing, the alignment errors of System I compared with System II are presented in Figure 9, and the installation error of the two systems was already taken into account. The experiment result indicates that the horizontal errors are about 0.04°, and the azimuth error is about 0.06°. Compared with the simulation results, it demonstrates that the performance of the horizontal attitude is worse, while the azimuth error is in accordance with the simulation. Further, the detailed graph shows that there is somewhat of an angle drift in the post-alignment period. The poor performance of pitch and roll, along with the drift of the attitude, could attribute to the misalignment between the odometer and the SINS. The wheel slip could also impact the velocity calculation, and therefore affect the alignment results. However, the azimuth result is far superior to the theoretical accuracy of the SINS with the applied gyros and the accelerometers. Furthermore, the alignment time is confined to 100 s, which is shorter than most marching alignment procedures, and especially important in motion operation for the vehicles.

**Figure 10:**
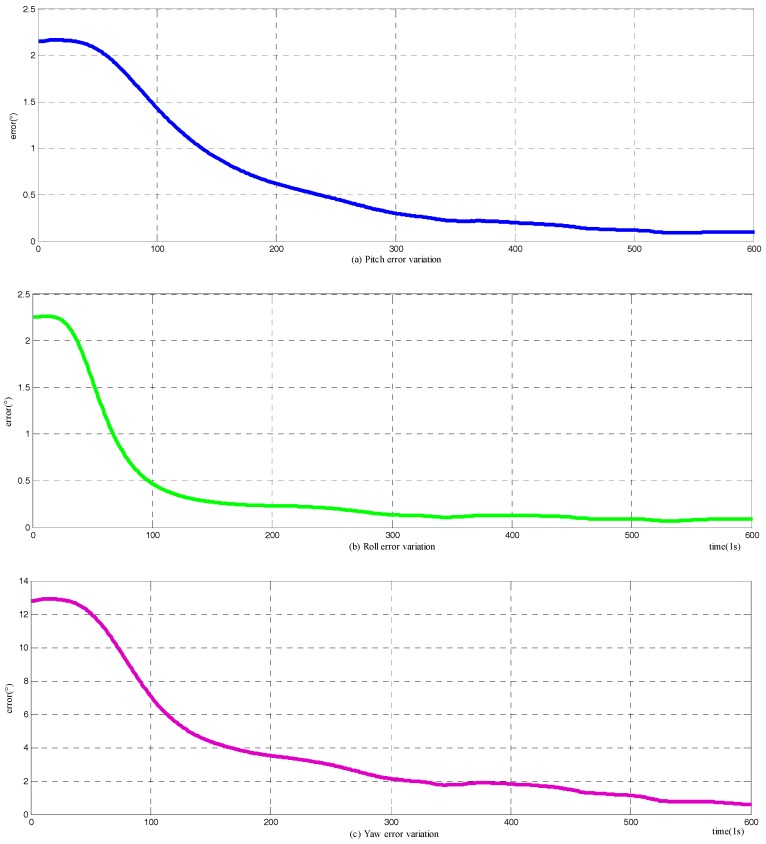
Attitude errors of the traditional alignment method in moving vehicle test: (**a**) the error curves of pitch; (**b**) the error curves of roll; (**c**) the error curves of yaw.

The comparison with the traditional alignment method could be carried out with the offline sensors data. The most frequently used alignment method for marching carriers is the fine alignment based on Kalman filter along with the coarse inertial alignment. This method could be described as having two stages: coarse inertial alignment period, consuming about 3 min; fine alignment based on Kalman filter, continuing calculation until the convergence of the attitude finished. With the same sensor data, the alignment processing was conducted, and the alignment errors relating to System II are presented in Figure 10. The coarse alignment did have a relatively large misalignment, and the fine alignment could converge the attitude to a smaller value. The convergence time for the attitude is a long period. There must be ~300–400 s for the attitude to approach the final value. The alignment accuracy is about 0.08° for horizontal angles, and 0.8° for the azimuth, which is worse than the theoretical value because of the dynamics.

**Figure 11:**
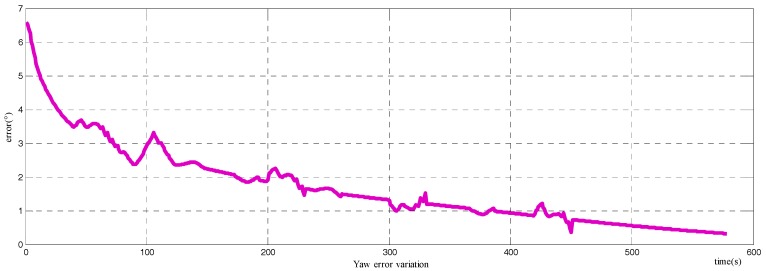
Yaw errors of the alignment method in [27].

Another comparison is also carried out with the alignment method proposed in [27], where the alignment is accomplished through the velocity vector from the GPS and integrated accelerations. As stated in [27], the method is more suitable when the carriers are marching with an approximate circumference motion. While for linear moving with turning in the heading of the carrier, the performance of the method is not ideal: the alignment time is long with low precision. The experiment result for the azimuth is shown in Figure 11. The alignment result does vary in the alignment period, and the final error of the azimuth is about 0.5°.

With the testing result of the novel alignment method and the comparison with typical methods, it can be concluded that: the novel alignment method could achieve an initial attitude establishment; it could accomplish the alignment within 100 s, which is much shorter than the other methods; the horizontal accuracy could achieve 0.03°, and the azimuth precision could achieve 0.06°. As a result, the novel method is suitable for situations when the carriers are in motion and it requires that the odometer should be calibrated in advance. However, the changing in the carrier environment could impact the performance of the method, which is necessary for further researching.

## 5. Conclusions

Tackling the requirement of fast initial alignment for a marching vehicle, a novel alignment procedure is proposed for the SINS aided by the GPS and the odometer. The matrix $C_{i_{0}}^{i_{b0}}$, the core problem of the computing of the attitude matrix $C_{b}^{n}$, can be achieved through the nonlinear velocity vectors information from the GPS and the odometer at different moments, denoted as the multi-vector attitude determination. Comparing with the existing alignment methods, this approach does not use the measurement information of accelerometers, while the velocity of the vehicle in the n frame and the b frame is needed synchronously. Compared with the existing alignment methods for a marching vehicle, the initial alignment can be fulfilled in shorter time, and does have superior performance, especially with relatively low-precision inertial sensors. Besides, the only requirement of this method for a marching vehicle is a turning movement in the alignment stage, which can be easily realized in the real conditions. The qualitative relation between measurement errors and alignment errors is given, and the alignment results are mostly impacted by the velocity from the GPS and the odometer. To cope with the random noise of velocity from the GPS, an improved method is put forward based on the error equation. A comparison with the two-position alignment method is developed to demonstrate its performance. According to the theoretical analysis, the simulation, and the vehicle test, the initial alignment can be fulfilled in 100 s, and the alignment azimuth error is smaller than 0.06° with the condition that the bias and random errors of gyros are 0.1° and 0.01°, respectively. Compared with the two-position alignment method, the accuracy of the horizontal angle is slightly worse, while the azimuth is much better and the alignment time is shorter with the same condition of gyros and accelerometers, particularly low-precision inertial sensors. The novel alignment method proposed could be applied in many fields where the carriers need to fulfill the alignment in a marching process. The application could include chariots, warships, and submarines, which are always in moving condition as a form of protection.

## Figures and Tables

**Table 1:** Sensor errors settings.

**Gyro**	Bias	0.1 °/h
	Random Walk	$0.01\;{}^{{}^{\circ}}/\sqrt{h}$
	Updating Frequency	1 kHz
**Accelerometer**	Bias	500 μg
	Updating Frequency	1 kHz
**GPS**	Velocity accuracy	0.03 m/s (RMS)
	Horizontal position accuracy	2 m (RMS)
	Updating Frequency	10 Hz
**Odometer bias**	Scale Factor	0.2%
	Updating Frequency	10 Hz

**Table 2:** Statistics of attitude error for alignment and calculation.

Statistics		Pitch (°)	Roll (°)	Yaw (°)
Mean	Alignment error	−0.0036	−0.1516	−0.0969
	Calculation error	−0.0036	−0.1497	−0.0065
Std	Alignment error	0.2823	0.0019	0.1574
	Calculation error	0.2819	9.8 × 10^−4^	0.3137

**Table 3:** Sensor errors of the high-precision strap-down inertial navigation system (SINS).

**Gyro**	Bias	0.01 °/h
	Random Walk	$0.001\;{}^{{}^{\circ}}/\sqrt{h}$
	Updating Frequency	1 kHz
**Accelerometer**	Bias	50 μg
	Updating Frequency	1 kHz
